# Purification of Sewage

**Published:** 1897-04-17

**Authors:** 


### PURIFICATION OF SEWAGE.

Mr. Fredk. J. Commin, surveyor to the Septic Tank System of Sewage Treatment, writes: In your issue of the 10th, on page 32, under the head "Purification of Sewage," you state that the Local Government officials have withheld their sanction to this system being adopted at Rushden and Staines. This is not in accordance with fact, as at neither place has any application to the Local Government Board been made, to our knowledge, for the adoption of the system, and I should esteem it if you would put this right in your next issue, as the statement is likely to do us injury.

